# Supplementary material for: A Comparative Study of Skeletal and Dental Outcome between Transcutaneous External Maxillary Distraction Osteogenesis and Conventional Rigid External Device in Treating Cleft Lip and Palate Patients
Source: J Pers Med. 2022 Jun 29;12(7):1062. doi: 10.3390/jpm12071062 (PMC9324202; doi:10.3390/jpm12071062)
Supplement: Supplementary file 1 [file jpm-12-01062-s001.zip › jpm-1791255-supplementary.pdf]

Table S1. Definition of landmarks and reference lines (according to Gao, 2022 and Lin, 2012).

Parameters	Abbreviation	Definition
Skeletal and dental landmarks		
Sella	S	Centre of the sella turcica
Nasion	N	The most anterior point of the nasofrontal suture in the median plane
Orbitale	Or	The lowest point on the average of the right and left orbital margins
Porion	Po	The most superior point on the average of bony external acoustic meatus
Subspinale	Point A	The deepest midline point in the curved bony outline from the base to the alveolar process of the maxilla
Supramentale	Point B	The most posterior point in the outer contour of the mandibular process in the median plane
Anterior nasal spine	ANS	The tip of the anterior nasal spine
Posterior nasal spine	PNS	The tip of the posterior nasal spine
Menton	Me	The most caudal point in the outline of the symphysis
Gonion	Go	The most posterior inferior point on the angle of the mandible
Incisor superius	U1i	The tip of the crown of the most anterior maxillary central incisor
Apex of upper incisor	U1a	The root apex of the most anterior maxillary central incisor
Soft tissue landmarks		
Pronasale	PRN	The most anterior, inferior point on the nose tip, which is intersected by a tangent line connecting with the chin profile
Soft A’ point	Soft A’ point	The most posterior point on the concavity between the upper lip and nose

Reference lines		
Frankfort horizontal plane	FH plane	Plane constructed by Po and Or
Nasion perpendicular line	Nperp	Nv Vertical reference to FH plane and passing through Nasion. Points anterior to Nperp are given a *positive* value, while posterior to Nperp are assigned a *negative* value
